# How Are We Educating Future Physicians and Pharmacists in Pakistan? A Survey of the Medical and Pharmacy Student’s Perception on Learning and Preparedness to Assume Future Roles in Antibiotic Use and Resistance

**DOI:** 10.3390/antibiotics10101204

**Published:** 2021-10-03

**Authors:** Naeem Mubarak, Sara Arif, Mahnoor Irshad, Rana Muhammad Aqeel, Ayesha Khalid, Umm e Barirah Ijaz, Khalid Mahmood, Shazia Jamshed, Che Suraya Zin, Nasira Saif-ur-Rehman

**Affiliations:** 1 Lahore Medical & Dental College, University of Health Sciences, Lahore 54600, Pakistan; mahnoorirshad6901@gmail.com (M.I.); ranaaqeel1996@gmail.com (R.M.A.); ayeshakhalid294@gmail.com (A.K.); bariraaijazz@gmail.com (U.eB.I.); dean.lpc@lmdc.edu.pk (N.S.-u.-R.); 2 Jinnah Burn and Reconstructive Surgery Center, Allama Iqbal Medical College, Lahore 54000, Pakistan; saraarif@jbrsc-aimc.edu.pk; 3 Institute of Information Management, University of the Punjab, Lahore 54000, Pakistan; khalid.im@pu.edu.pk; 4 Department of Clinical Pharmacy and Practice, Faculty of Pharmacy, Universiti Sultan Zainal Abidin, Kuala Terengganu 22200, Malaysia; 5 Kulliyyah of Pharmacy, International Islamic University Malaysia, Kuantan 25200, Malaysia

**Keywords:** antibiotic resistance, pharmacy education, medical education, educational policy, antibiotic use, Pakistan

## Abstract

Background: Medical and pharmacy students are future healthcare professionals who will be on the forefront in dealing with antibiotics in hospitals or community settings. Whether the current medical and pharmacy education in Pakistan prepares students to take future roles in antibiotic use remains an under-researched area. Aim: This study aims to compare medical and pharmacy students’ perceived preparedness, learning practices and usefulness of the education and training on antibiotic use and resistance imparted during undergraduate studies in Pakistan. Design and Setting: It was amulti-centre cross-sectional survey of medical and pharmacy colleges in Punjab, Pakistan. Method: A self-administered questionnaire was used to collect data from final year medical and pharmacy students. Descriptive statistics were used for categorical variables while independent *t*-test and One-way ANOVA computed group differences. Result: Nine hundred forty-eight respondents (526 medical and 422 pharmacy students) completed the survey from 26 medical and 19 pharmacy colleges. Majority (76.1%) of the pharmacy students had not completed a clinical rotation in infectious diseases. The top three most often used sources of learning antibiotic use and resistance were the same among the medical and the pharmacy students; included textbooks, Wikipedia, and smart phone apps. Overall self-perceived preparedness scores showed no significant difference between pharmacy and medical students. The least prepared areas by medical and pharmacy students included transition from intravenous to oral antibiotics and interpretation of antibiograms. Both medical and pharmacy students found problem solving sessions attended by a small group of students to be the most useful (very useful) teaching methodology to learn antibiotic use and resistance. Conclusions: Differences exist between medical and pharmacy students in educational resources used, topics covered during undergraduate degree. To curb the growing antibiotic misuse and resistance, the concerned authorities should undertake targeted educational reforms to ensure that future physicians and pharmacists can play a pivotal role in rationalizing the use of antibiotics.

## 1. Background

Antibiotic resistance—the capability of bacteria to surpass the effects of antibiotics—has emerged as one of the top ten challenges of 21st century. It means the resource pool of antibiotics will lose its effectiveness leading to its partial or complete depletion. Nowadays, antibiotic resistance (ABR) is termed a “tragedy of commons” because of the irreversible fallouts on society including incurable or long-standing common infections and significant rise in mortality, healthcare cost and hospital stays [1]. 

A growing body of evidence warns of the dramatic spread of ABR in low- and middle-income countries (LMICs) in the Asian region [2]. One of the established reasons behind this continuous surge in bacterial resistance is pervasive misuse of antibiotics in hospitals and community settings in Asia. This misuse of antibiotics emanates from fallacies in the choice of antibiotic, posology, route of administration, duration of therapy, over prescribing and unauthorized sale of antibiotics [2,3]. Multiple factors cause the malpractices of overprescribing and sale of antibiotics without prescription; however, inadequate knowledge and training of health care professionals have been dubbed as the *fons et origo* of the two malpractices [3]. 

The World Health Organization’s Global Action Plan emphasizes the education and training of the prescriber and other healthcare professionals as one of five priority interventions to clampdown growing bacterial resistance and optimize the use of antibiotics [4]. Education of healthcare professionals is a continuous process, however, the undergraduate level of medical, pharmacy or any other allied health discipline is the first step to acquire related knowledge and build a strong foundation for any further specialty [5]. Notably, medical and pharmacy students are future healthcare professionals who will be dealing with antibiotics in hospitals, primary care and community settings. On one hand, medical students will be the future prescribers, and on the other hand, pharmacy students will advise on the rational use of antibiotics in hospitals at the conveniently accessible location of a community pharmacy somewhere [6,7]. Hence, future management of the challenges associated with bacterial resistance or misuse of antibiotics depends upon the quality of education, training and skills learnt at an undergraduate level and this accentuates the pivotal value of undergraduate studies for the upcoming years. Nevertheless, during early education of health care professionals, different countries deploy different pedagogical approaches to teach optimal antibiotic use [7].

Pakistan, an LMIC in South Asia, is an important region for interventions to reduce the growing ABR owing to its huge population of 216 million inhabitants [8]. Officially, there are 62 medical colleges (19 public, 43 private) in Punjab and Islamabad [9]. The Pakistan Medical Commission (PMC), formerly known as Pakistan Medical & Dental Council, and the Higher Education Commission (HEC), are the regulatory bodies with a mandate to set and implement a uniform curriculum for the five-year degree program Bachelor of Medicine and Bachelor of Surgery (MBBS), as well as accrediting medical colleges across the country. Meanwhile, The Pharmacy Council of Pakistan (PCP) accredits pharmacy colleges, registers pharmacists, and sets standards of pharmacy education and training across the country. A five-year curriculum, developed and approved by the HEC in consultation with PCP, leads to the degree of Doctor of Pharmacy (Pharm D). At the moment, 49 pharmacy colleges (10 public, 39 private) are approved by the PCP to offer a Pharm D degree program in Punjab [10]. 

Studies have warned of pervasive overprescribing by physicians in hospitals and the sale of antibiotics without prescription in community settings in Pakistan [11]. Significant gaps exist in the continuous education of healthcare professionals on optimal antibiotic use and growing resistance in tertiary care hospitals [8,12]. Whether these gaps originate from a lack of training and education at the beginning (during the undergraduate degree program, such as MBBS or Pharm D) or during professional practices, remains a debatable issue. Meanwhile, different studies report sub-optimal knowledge on the part of pharmacy and medical students regarding antibiotics use, resistance, and stewardship [13]. Despite antibiotic resistance being a significant threat in Pakistan, few studies have shed light on the nature of the contents of the training and education that students have undergone during MBBS and Pharm D degrees in Pakistan. Nevertheless, hardly any study has evaluated and compared preparedness or perception of the quality of medical and pharmacy education and training. Thus, an in-depth analysis of the quality of training and education at an undergraduate level is notably absent. A greater understanding is required about how the principals of appropriate antibiotics use are taught, at which level students are introduced to these concepts, and whether teaching made students confident enough to translate these concepts into practice. Therefore, this study aims to understand the perception of medical and pharmacy students about their education and training imparted during undergraduate studies on the appropriate antibiotic use and resistance in Pakistan. The findings of this study will recognize current trends in academic practices in medical and pharmacy colleges and may be used to urge action to improve future teaching practices and curriculum design for pharmacy and medical undergraduate degree programs. 

## 2. Methods

### 2.1. Study Design, Sample and Setting

A cross-sectional, multicentre survey was designed to collect data of final year MBBS and Pharm D students in medical and pharmacy colleges in the province of Punjab and Islamabad Capital Territory in Pakistan. Lists of the approved medical and pharmacy colleges were obtained from the official websites of PMC and PCP respectively.

An online freely available software, Raosoft, calculated the sample size independently for pharmacy (*n* = 257) and for medical (*n* = 373) with a 5% margin of error, 95% confidence interval and assuming a maximum possible population of 20,000 for each discipline at a response distribution of 50%.

### 2.2. Survey Instrument, Data Collection and Analysis

The survey instrument was adopted from the questionnaire used in a previous study [14]. It was comprised of five sections in English language:

1. Demographics of the respondent (e.g., sex, name of college, ownership of college, previous training before the formal degree etc.).
2. The topics covered related to responsible antibiotic use and resistance during the undergraduate degree program.
3. The resources preferred by the students to learn about antibiotic use and resistance.
4. Perceived usefulness of different teaching methods deployed to teach antibiotic use and resistance.
5. Perceived preparedness of the students to take future roles in antibiotic use and resistance.

Researchers deployed four data collection teams, each one consisting of five Doctor of Pharmacy (final year) students. We trained data collection teams for two days on topics, such as aims of the study, items in the survey, possible queries, and ensuring completeness of the questionnaire filled. Data were collected on paper, self-administered, over a period of three months (17 October 2019, to 15 January 2020). After permission from the concerned authorities in the participating colleges, data collectors were escorted to final year students. Data collector teams first introduced the aims and objective of the study to students and then distributed the survey. Data on paper were later exported in Statistical Package Social Sciences SPSS (version 23 IBM, Armonk, NY, USA) for analysis. Categorical data were reported using frequencies and percentages, while continuous variables were described using mean and standard deviation. Student *t*-test was used to commute two group differences while one way ANOVA was used to evaluate more than two group differences. The rank order of different resources used by the students was computed based on the mean score value where the higher the mean score was, higher the rank (‘1’ = the highest).

### 2.3. Inclusion and Exclusion Criteria

Final year enrolled students from any medical or pharmacy college in Punjab and Islamabad were eligible to participate (subject to willingness) in the survey. Researchers surveyed final year students of both professions because students in final year have mainly completed all the subjects related to the topic of this study. 

Non-final year students in medical and pharmacy disciplines were excluded because they had not completed all the necessary subjects related to the topic of this study, hence, their experience might lead to a bias. Recently accredited/approved medical or pharmacy colleges were excluded because these colleges had initial classes and lacked final year students. Finally, the study excluded medical and pharmacy colleges which were not recognized by the PMC and PCP, respectively; because that indicated a lack of compliance to the minimum set standards of education and training.

### 2.4. Ethics

Research Ethics Committee, Lahore Pharmacy College, Lahore Medical & Dental College granted ethical approval to carry out this study (ref: ETH/LPC/15/07/19). Informed consent was taken from the respective administrative head of the participating college, after detailing them about the aims of the study and how the data would be used for publication in later stages. The research team assured the anonymity of data and responses during the publication process. For this purpose, the research team assigned individual identification numbers to colleges. Afterwards, data were stored in a password-protected computer.

## 3. Results

### 3.1. Demographic Data and Response Rate

A total of 948 respondents (526 medical and 422 pharmacy students) completed the survey from twenty-six medical colleges (institutional response rate 76.5%) and nineteen pharmacy colleges (institutional response rate. 70.40%). Based on the exclusion criteria, 20 pharmacy and 4 medical colleges were excluded. The mean age of the respondents was 23.3 years and majority of the respondents were females in both medical (53.2%) and pharmacy colleges (63%). A vast majority (76.1%) of the pharmacy students had not completed a clinical rotation in infectious diseases, compared with 57% of the medical students who reported completion of a clinical rotation in infectious diseases. Detailed demographics are provided in Supplementary file (Table S1).

### 3.2. Topics Covered on Responsible Antibiotic Use and Resistance

Disappointingly, a vast majority of the medical (83%) and pharmacy students (78%) were not familiar with the term antimicrobial stewardship. Less than half of the students in both disciplines had attended lectures on the rational use of antibiotics in general. Compared with medical students, more pharmacy students acknowledged receiving formal lectures on selection of the correct antibiotic dose and right duration of treatment for specific infections and the difference was statistically significant. Table 1 lists various topics covered during the degree programs.

### 3.3. Educational Resources

Both medical and pharmacy students reported a variety of resources for learning optimal antibiotic use and resistance. The top three most often used sources of learning antibiotic use and resistance were the same among the medical and the pharmacy students and included textbooks or study guides, followed by Wikipedia and smart phone apps. However, there was a considerable variability in ranking of medical and pharmacy students. For instance, peer learning was popular among medical students who ranked it the fourth most often used resource, while pharmacy students ranked consultation with the hospital/clinical pharmacist as the fourth most used source. Notably, neither pharmacy nor medical students preferred any kind of infectious disease guidelines (ranked 14th, the least consulted source) Table 2 compares the rankings of different sources of learning by medical and pharmacy students. 

### 3.4. Perceived Usefulness of Teaching Methodology

**Figure 1:**
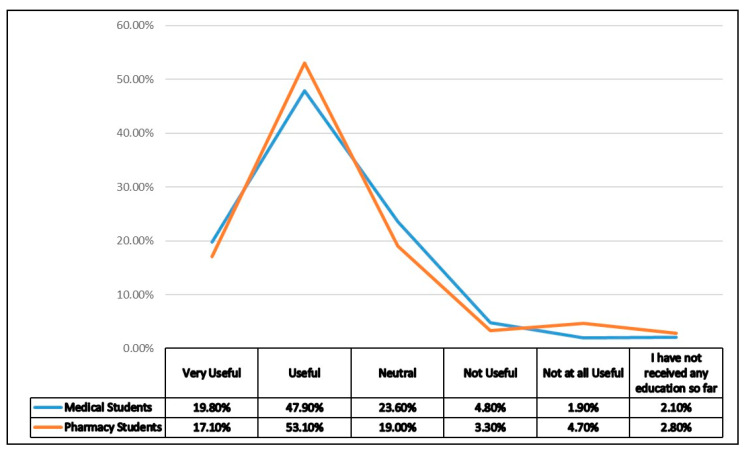
Overall usefulness of education regarding appropriate use of antimicrobials.

Both medical and pharmacy students found problem solving sessions attended by a small group of students as the most useful (very useful) teaching methodology to learn antibiotic use and resistance, followed by the grand round’s lectures i.e., discussion of clinical cases. Interestingly, classroom lectures were rated very useful by only 8.2% of medical and 11.1% of pharmacy students and thus indicate a lack of effectiveness. Table 3 contrasts the perceived usefulness of different teaching methods among the pharmacy and medical students in Punjab, Pakistan. Furthermore, as a whole, only 19.8% medical and 17.1% pharmacy students rated their education and training on appropriate use of antibiotics as very useful (Figure 1).

### 3.5. Perceived Preparedness

The overall mean score on perceived preparedness was higher among pharmacy students as compared to medical students; however, the difference was not statistically significant (*p* = 0.15). Among the pharmacy students, based on the mean score, the top three least prepared individual curriculum items included; interpretation of antibiograms (mean, 2.96, *p* = 0.48), followed by transition from intravenous to oral antibiotics (mean, 3.03, *p* = 0.04), and how to streamline or deescalate antibiotics therapy (mean, 3.09, *p* = 0.03) as mentioned in Table 4. Whereas, among the medical students, the top three topics perceived as least prepared included; lack of competency to decide the transition from intravenous to oral antibiotics (mean score, 2.91, *p* = 0.04) followed by how to select the best antimicrobial for a specific infection (mean, 2.94, *p* = 0.00), and how to interpret the antibiograms (mean, 3.02, *p* = 0.48) (Table 4). While, both medical and pharmacy students well understand the basic mechanisms of antimicrobial resistance (mean, 3.44 and 3.56 respectively, *p* = 0.06). Table 4 presents a comparison of the preparedness of medical vs. pharmacy students on various topics related to optimal antibiotic use. 

Finally, results of analysis of variance reveal that public medical colleges were better prepared as compared to private medical colleges and the difference was statistically significant. Supplementary file (Table S2) offers comparison of the mean of four subgroups in the sample.

## 4. Discussion

This study assessed medical and pharmacy students’ self-reported preparedness, usefulness of different learning methods, range of topics covered, and common educational resources preferred for antibiotic use and resistance. Our study noted several important findings: 

First, some major principles on prudent antibiotic use were perceived poorly prepared which implies various areas where both pharmacy and medical students require more training. For instance, medical students feel unprepared on the complexities in selection of the best antimicrobial for a specific infection in daily practice, transition from intravenous to oral, and finding a reliable source of information. These gaps in training during undergraduate studies may also be correlated with the shortcomings identified in antibiotics prescriptions in hospitals in Punjab where duration of antibiotic therapy or doses of antibiotics and switch from intravenous to oral or route of administration were absent or wrongly mentioned on the prescriptions [15,16]. A key consideration of these findings would be that the low prepared areas should now be prioritized for improvement during teaching planning. 

Second, in terms of practical exposure, a vast majority of pharmacy students report lack of clinical rotation in infectious diseases, which is worrying as it will limit the participation of pharmacists in any future antibiotic stewardship role [7]. A number of studies have mentioned participation in clinical rotation as the strongest predictor of greater knowledge in students and concluded that clinical rotation in infectious diseases improves knowledge of appropriate antibiotic use as compared to absentee students in clinical rotation [17,18]. 

Third, our findings reveal that passive forms of learning, such as classical classroom lectures were not perceived as useful or popular in today’s generation in Pakistan. Many universities have adopted problem based learning and improved students understanding. A number of studies support the idea that active/interactive forms of learning have higher and longer-term influence on prescribing habits later in professional life [19,20]. A study in Nepal highlighted the successful teaching of antimicrobials through a problem-solving approach as part of pharmacology lectures [20]. Furthermore, in terms of adult learning behaviour, it is always beneficial to teach concepts from a disease-oriented approach (e.g., urinary tract infections) or problem-oriented approach (e.g., antimicrobial resistance) rather than a drug-oriented approach (e.g., cramming classification of antibiotics) or a pathogen-oriented approach (e.g., methicillin-resistant Staphylococcus aureus) [5,18]. 

Many developed countries have recognized the importance of undergraduate training and education and its impact on professional practice and attitude. For instance, in the UK, a Specialist Advisory Committee on Antimicrobial Resistance has proposed a robust framework for developing curricula to learn the rational use of antibiotics [21]. This framework is based on developing core-learning outcomes (statements that define what a student should know or be able to perform by the end of an educational intervention). Teaching resources based on clinical case scenarios (vignettes) and problem-based learning would be used to ensure that the learning outcomes were achieved. The learning outcomes may be translated later into competencies that can be objectively measured by a concerned body for the accreditation purposes. Similarly, in the United States, a new curriculum on antimicrobial stewardship has been jointly designed by the U.S. Centres for Disease Control and Prevention and the Association of American Medical Colleges [5,21]. While, in Pakistan, conversion of a Bachelor of Pharmacy (a four years degree program) to Doctor of Pharmacy (a five years degree program) happened in 2003 with an aim to inculcate a more patient-oriented (clinical) curricula to pharmacy students. Pharm D curricula was last updated in 2013, however, the clinical focus remained deficient and our study also reports that a vast majority of the students have not received any clinical rotation in infectious diseases [10].

Fourth, a vast majority of medical and pharmacy students were not familiar with the term antibiotic stewardship. This finding is in line with the results reported in the study by Hayat et al. [13]. Antibiotics use, resistance and mechanisms are part of the curriculum of both Pharm D and MBBS degree programs, however, specifically, principals of antibiotics stewardship and its concept is relatively new and grossly absent in both curricula. 

Fifth, although the top three learning resources used in both disciplines were more or less the same, both medical and pharmacy students did not consult any professional guidelines as a preferred source of learning rational antibiotic use. This behaviour may have resulted from a lack of focus on guidelines within the curricula, which does not bind students or teachers to develop these habits. Research has shown that students who use the Infectious Diseases Society of America (IDSA) guidelines demonstrate better and structured knowledge of antibiotic use than those who do not use guidelines. Furthermore, students who consulted guidelines during undergraduate studies are more likely to practice rational use of antibiotics later in professional practice [14,22]

Sixth, lack of inter-professional collaboration between medical and pharmacy students was evident. Students did not prefer to consult pharmacist or infectious disease physician for knowledge exchange, even though both disciplines will assume the future role of a patient educator, especially the pharmacist working in a community pharmacy, and will formidably influence consumer behaviour on irrational and unauthorized purchase of antibiotics without prescription. 

Finally, various studies have concluded that it is easier to shape behaviours during early education and training than changing established habits later during professional life [5,22]. While, traditionally, a major focus of education has been on the experienced prescribers and pharmacists, students or residents received little attention. This despite the idea that they may otherwise benefit more from educational interventions, being in an early phase of habit development. Research supports the idea that better training of students and residents may result in long lasting antibiotics stewardship habits [19]. Furthermore, for a resource limited setting i.e., low- and middle-income countries, a focus on education may be the least resource consuming interventions to reduce antibiotic overuse [3].

### 4.1. Comparison with Existing Literature 

The findings regarding the choice of educational resources by medical and pharmacy students reported in our study are in line with a previous study conducted in Punjab [23]. Both studies found that medical and pharmacy students prefer to use textbooks to learn antibiotic use. However, there exists methodological differences in terms of data analysis in addition to the larger and diverse sample size in our study which may yield a comparatively more reliable and generalizable result. However, our findings regarding self-perceived preparedness are not in consonance with the mentioned study that reported no significant difference between pharmacy and medical students except on the statement that asked to describe the correct spectrum of antimicrobial therapy. These differences in findings may be linked with the lack of diversity or inclusiveness in terms of the number of colleges in the sample recruited to report the results (only two institutions participated in the study). While we reported mixed findings and concluded significant differences in preparedness between medical and pharmacy students based on a diverse sample. Pharmacy students were poorly prepared in how to de-escalate antibiotic therapy and transition from intravenous antibiotics to oral. While pharmacy students were better prepared to find a reliable source of information to treat infections, and to select the best antimicrobial. Interestingly, these findings are in line with the results of a multicentre survey conducted in three Asian countries (Indonesia, Malaysia, and Pakistan) that reported similar results on self-perceived confidence of pharmacy students to perform the tasks mentioned [7]. Another study in Bangladesh reported similar lacks in the preparedness of medical students on antibiotic use and resistance as reported in our study and suggested an upgrade of curricula on antimicrobial stewardship as educational interventions [24].

A recent study conducted in seven pharmacy institutes in Pakistan concluded average knowledge of the students on certain aspects of the antibiotic use and only 21.6% of students were familiar with the antibiotic stewardship program [13]. These results are in line with the results of our study that involved a comparatively larger sample. However, our study contradicts the findings of this study that report that public university students had a superior knowledge and reported no significant differences between the knowledge of pharmacy students from private or public institutions in any contexts. The possible reason could be the different statements used to develop a construct to cover antibiotic use and resistance. 

Comparing at a regional level, a multicentre survey in five Asian countries (Malaysia, Thailand, Singapore, Indonesia, and the Philippines), reports that 87% of the schools use interactive teaching formats and mainly deliver the knowledge through small group discussion (93%) vs. clinical case studies (69%), lectures (69%) and role-play (21%). Interactive learning has improved many aspect of student learning on antibiotic use [25]. This state of affairs implies that in the region, various other countries are steps ahead as compared to Pakistan in terms of teaching methods. While, our study reports pervasive classical lectures format and less interactive learning in both medical and pharmacy schools. Another study conducted in central China revealed similar gaps in the teaching of medical education to those in our study and highlighted the need for educational reform on medical education in developing countries. Particularly, our results in the educational resources used among medical students in Pakistan are quite similar and indicate the need for a change in the teaching practices in the region [26].

### 4.2. Implications for Policy and Practice and Further Research

Based on the findings, the current study suggests the following implications for policy, practice, and further research:

First, in order to boost students’ knowledge of optimal antibiotic use and prescribing; reforms in the concerned undergraduate curriculum are inevitable. Curricula are being revised worldwide; hence, a strong foundation within the existing curriculum is crucial to prepare future healthcare professionals who can rationalize antibiotic use in hospitals and in the community. It is time that PMC and PCP also revise respective curricula in consultation with HEC toward antibiotic stewardship and optimal antibiotic use. For this, the authors suggest integrating dedicated modules on antibiotic stewardship in the subject of Pharmacology or Clinical Pharmacy. Second, clinical rotation in infectious diseases should be a priority in future curricula reforms especially in the pharmacy. Third, given the gaps in teaching methods, curricula should focus more on active (interactive) learning techniques such as patient case studies and problem-based learning involving small groups to deliver key concepts of prudent antibiotic use. Fourth, the addition of inter-professional workshops right at the start of undergraduate training to overcome future barriers in working as a team.

Fifth, there should be a change in educational resources and students should practice the use of standard guidelines. Finally, there is a crucial need for further research to understand how students’ self-perceived preparedness reflects observed preparedness and how this in turn translates into clinical practice. A natural extension of this study may involve a systematic comparison of the formal medical and pharmacy curricula and pedagogical and assessment methods used in medical and pharmacy colleges in Pakistan with internationally agreed upon sets of competencies in antimicrobial stewardship to identify and address potential lacunas in training. Meanwhile, we expect that our results provide useful insight for faculty currently teaching the rational use of antibiotics to pharmacy and medical students in Pakistan.

### 4.3. Limitations and Strengths of the Study

We noted the following limitations/strengths of the study.

(a) Our study did not recruit students or colleges based on random sampling; however, we do not believe it will have introduced significant selection bias given the topic of the study and the lack of incentives to participate. Moreover, this study collected data from a majority of the medical and pharmacy colleges in the province and thus we anticipate that results are likely to represent the overall scenario in the country.
(b) Colleges under the auspices of Pakistan Armed Forces were not included in the sample because a time taking procedure to seek permission was required from General Headquarters. However, based on the diversity of the sample, since students participated from a large majority of the colleges, results appear to be generalizable and more likely to represent the overall scenario in the country.
(c) Another limitation of this study was assessment bias. The questions in the survey may not have necessarily covered all the dimensions of preparedness or educational resources or teaching practices on antibiotic resistance, antibiotic therapy, and antibiotic stewardship.
(d) As some of the questions required the respondents to recall certain information, the possibilities of the risk of recall bias could be there.

#### Strengths

(a) To our knowledge, this is the first comprehensive comparative study to report current practices and perceived preparedness of the medical and pharmacy students on antibiotic use.
(b) Our study was free of recruitment bias as the data collectors did not opt for a convenient sample of the colleges but included medical and pharmacy colleges in nine administrative divisions of Punjab Pakistan.
(c) Furthermore, since students participated from nearly all medical and pharmacy colleges results likely to be highly relevant to all institutions in the country.
(d) Study reported specific gaps in teaching, and identified poorly prepared areas related to prudent antibiotic use.

## 5. Conclusions

Significant differences exist between medical and pharmacy students in educational resources used, topics covered during undergraduate degrees, and perceived preparedness to take future roles in antibiotic use. Both medical and pharmacy students demonstrated low self-perceived preparedness and require more training in selection of antibiotic, posology, duration and de-escalation and transition from intravenous to oral delivery of the antibiotic therapy. Both medical and pharmacy students did not find lecture-based learning useful for learning antibiotics use. There is a strong reason for action and we urge educational policy makers in Pakistan to take a step forward to upgrade the decade old curricula of the Pharm D and MBBS degree program to one that uses problem-based learning with increased practical exposure. This sort of education should start at an early stage, during the undergraduate curriculum, to ensure maximal effect and to foster the right attitudes.

The problem does exist at the beginning at least in this part of the world, hence, scientists alone cannot tackle the looming crisis of antibiotic resistance, and we need educationist too. To curb growing antibiotic misuse and resistance, the concerned stakeholders in Pakistan should undertake targeted educational reforms to ensure that medical and pharmacy students leave the college with confidence to play a pivotal role in rationalizing the use of antibiotics in hospitals and community settings.

## Figures and Tables

**Table 1:** Have you attended any formal lecture(s) that address the following topics during medical/pharmacy College?

Statements	Status	No *n* (%)	Yes *n* (%)	I don’t Remember *n* (%)	χ2 (*p*-Value)
Rational use of antibiotics in general	P	192 (45.5)	199 (47.2)	31 (7.3)	1.79
	M	218 (41.4)	262 (49.8)	46 (8.7)	(0.408)
When to start antibiotics	P	112 (26.5)	265 (62.8)	45 (10.7)	3.51
	M	169 (32.1)	306 (58.2)	51 (9.7)	(0.173)
How to select the correct dosage?	P	119 (28.2)	257 (60.9)	46 (10.9)	18.62
	M	217 (41.3)	252 (47.9)	57 (10.8)	(0.000) *
How to select the duration of treatment for specific infections?	P	125 (29.6)	237 (56.2)	60 (14.2)	7.26
	M	199 (37.8)	266 (50.6)	61 (11.6)	(0.027) *
Are you familiar with the term antimicrobial stewardship (AMS)?	P	331 (78.4)	37 (8.8)	54 (12.8)	9.03
	M	437 (83.1)	52 (9.9)	37 (7.0)	(0.011) *

*n* = number, % = percentage, χ2 = Chi-square test, P = pharmacy students, M = medical students. * *p* ≤ 0.05 (significant difference).

**Table 2:** How do you use each of the following sources to learn about antimicrobials use and resistance?

Statements	Medical Students		Pharmacy Students		*t*
	Mean (SD)	Rank	Mean (SD)	*Rank*	(*p*-Value)
Textbooks or study guides	3.17 (0.95)	1	3.24 (0.89)	*1*	1.23
					(0.221)
Wikipedia	3.01 (0.98)	2	3.07 (0.95)	*3*	0.19
					(0.849)
iPhone or smartphone apps	2.94 (1.02)	3	3.02 (0.99)	*2*	2.13
					(0.034) *
Peers (other students)	2.89 (1.05)	4	3.01 (0.91)	*7*	−0.21 **
					(0.833)
Others used often	2.89 (1.08)	4	2.96 (1.01)	*6*	0.01
					(0.996)
Up to Date	2.87 (1.01)	5	2.89 (1.12)	*5*	1.38
					(0.168)
Infectious Diseases specialists	2.86 (1.02)	6	2.87 (1.04)	*7*	0.20
					(0.844)
Hospital/ clinical Pharmacists	2.76 (0.97)	7	2.87 (0.97)	*4*	4.13
					(0.000) *
Medical Journals	2.67 (0.99)	8	2.86 (1.02)	*9*	2.39
					(0.017) *
Other Guidelines by professional organizations	2.63 (1.00)	9	2.83 (0.99)	*10*	2.96
					(0.003) *
Non-infectious diseases physicians	2.61 (0.97)	10	2.82 (0.98)	*14*	−1.58 **
					(0.113)
Pharmaceutical representatives	2.58 (1.05)	11	2.68 (1.08)	*8*	4.02
					(0.000) *
Johns Hopkins Antibiotic guide	2.57 (1.08)	12	2.59 (1.14)	*12*	0.24
					(0.811)
Sanford guide	2.55 (1.08)	13	2.55 (1.14)	*13*	−0.02 **
					(0.988)
Infectious Diseases Society of America Guidelines	2.53 (1.00)	14	2.50 (0.99)	*11*	2.20
					(0.027) *

SD = standard deviation, *t* = independent *t*-test. * *p* ≤ 0.05 (significant difference).** In statistics, a negative *t*-value elaborates that the direction of the effect under study is being reversed; this value has no impact on the significance of difference between groups of data. Pharmacy students’ rank was italicized to differentiate from the rank given by the medical students.

**Table 3:** If made available to you, please rate the usefulness of each of the following options for learning about antimicrobial use and resistance?

Statements	Status	Not at all Useful *n* (%)	Not Useful *n* (%)	Neutral *n* (%)	Useful *n* (%)	Very Useful *n* (%)	Median (IQR)
Grand rounds lecture	P	18 (4.3)	41 (9.7)	126 (29.9)	154 (36.5)	83 (19.7)	4 (1)
	M	43 (8.2)	53 (10.1)	100 (19.0)	182 (34.6)	148 (28.1)	4 (2)
Classroom lectures	P	28 (6.6)	206 (48.8)	85 (20.1)	56 (13.3)	47 (11.1)	2 (1)
	M	45 (8.6)	227 (43.2)	21 (4.0)	190 (36.1)	43 (8.2)	2 (2)
Lecture series of medical and pharmacy students.	P	29 (6.9)	34 (8.1)	116 (27.5)	161 (38.3)	82 (19.4)	4 (1)
	M	39 (7.4)	75 (14.3)	182 (34.6)	154 (29.3)	76 (14.4)	3 (1)
Interactive patient oriented problem-solving modules on the internet.	P	30 (7.1)	41 (9.7)	156 (37.0)	118 (28.0)	77 (18.2)	3 (1)
	M	35 (6.7)	60 (11.4)	171 (32.5)	134 (25.5)	126 (24)	3 (1)
Interactive patient oriented problem-solving modules on CD-ROM.	P	38 (9.0)	46 (10.9)	138 (32.7)	147 (34.8)	53 (12.6)	3 (1)
	M	33 (6.3)	70 (13.3)	163 (31.0)	57 (29.8)	103 (19.6)	3 (1)
Problems solving sessions attended by small groups of medical/pharmacy students and residency or faculty.	P	25 (5.9)	39 (9.2)	84 (19.9)	136 (32.2)	138 (32.7)	4 (2)
	M	31 (5.9)	55 (10.5)	100 (19.0)	185 (35.2)	155 (29.5)	4 (2)
Role playing sessions dealing with patients demanding antimicrobial therapy.	P	28 (6.6)	40 (9.5)	155 (36.7)	121 (28.7)	78 (18.5)	3 (1)
	M	59 (11.2)	97 (18.4)	90 (17.1)	142 (27.0)	138 (26.2)	4 (3)

*n* = number, % = percentage, IQR = interquartile range, P = pharmacy students, M = medical students.

**Table 4:** How well do you feel that your medical/pharmacy education has prepared you to do the following upon graduation?

Statements	Medical Students	Pharmacy Students	*t* (*p*-Value)
	Mean (SD)	Mean (SD)	
To know when to start antimicrobial therapy	3.30	3.36	1.01
	(0.97)	(0.99)	(0.314)
How to select the best antimicrobial for a specific infection?	2.94	3.18	0.28
	(1.13)	(1.03)	(0.001) *
To describe the correct spectrum of antimicrobial therapy for different antimicrobials (what is covered by each drug)	3.32	3.28	−0.51 **
	(1.00)	(1.04)	(0.613)
Understand the basic mechanisms of antimicrobial resistance	3.44	3.56	1.89
	(1.01)	(0.97)	(0.059) *
How to streamline or de-escalate antimicrobial therapy?	3.23	3.09	−2.24 **
	(0.99)	(0.95)	(0.025) *
How to interpret antibiograms?	3.02	2.96	−0.71 *
	(1.11)	(1.10)	(0.481)
How to find reliable sources of information to treat infections?	3.24	3.42	2.68
	(1.05)	(1.01)	(0.008) *
How to transition from intravenous to oral antibiotics (IV to PO switch)?	2.91	3.03	2.02
	(0.96)	(0.88)	(0.044) *
How to handle a patient who demands antimicrobial therapy that is not indicated?	3.23	3.31	1.12
	(1.13)	(1.07)	(0.262)
Overall Score	3.18	3.24	1.44
	(0.70)	(0.68)	(0.152)

SD = standard deviation, *t* = independent *t*-test, IV = intravenous, PO = per oral. * *p*≤ 0.05 (significant difference). ** In statistics, a negative *t*-value elaborates that the direction of the effect under study is being reversed, this value has no impact on the significance of difference between groups of data.

## Data Availability

Research team has made all the underlying data available to the public in the supplementary files. Furthermore, the corresponding authors may be contacted for any further clarifications.

## Supplementary Materials

The following are available online at https://www.mdpi.com/article/10.3390/antibiotics10101204/s1, Table S1: Demographics of respondents, Table S2: How well do you feel that your medical/ pharmacy education has prepared you to do the following upon graduation?

## References

[1] Dhingra S., Rahman N.A.A., Peile E., Rahman M., Sartelli M., Hassali M.A., Islam T., Islam S., Haque M. (2020). Microbial Resistance Movements: An Overview of Global Public Health Threats Posed by Antimicrobial Resistance, and How Best to Counter. Front. Public Health.

[2] Yam E.L.Y., Hsu L.Y., Yap E.P.-H., Yeo T.W., Lee V., Schlundt J., Lwin M.O., Limmathurotsakul D., Jit M., Dedon P. (2019). Antimicrobial Resistance in the Asia Pacific region: A meeting report. Antimicrob. Resist. Infect. Control..

[3] Godman B., Egwuenu A., Haque M., Malande O., Schellack N., Kumar S., Saleem Z., Sneddon J., Hoxha I., Islam S. (2021). Strategies to Improve Antimicrobial Utilization with a Special Focus on Developing Countries. Life.

[4] Munkholm L., Rubin O. (2020). The global governance of antimicrobial resistance: A cross-country study of alignment between the global action plan and national action plans. Glob. Health.

[5] Nogueira-Uzal N., Zapata-Cachafeiro M., Vázquez-Cancela O., López-Durán A., Herdeiro M.T., Figueiras A. (2020). Does the problem begin at the beginning? Medical students’ knowledge and beliefs regarding antibiotics and resistance: A systematic review. Antimicrob. Resist. Infect. Control.

[6] Marzan M., Islam D.Z., Lugova H., Krishnapillai A., Haque M., Islam S. (2021). Knowledge, attitudes, and practices of antimicrobial uses and resistance among public university students in Bangladesh. Infect. Drug Resist..

[7] Abubakar U., Muhammad H.T., Sulaiman S.A.S., Ramatillah D.L., Amir O. (2020). Knowledge and self-confidence of antibiotic resistance, appropriate antibiotic therapy, and antibiotic stewardship among pharmacy undergraduate students in three Asian countries. Curr. Pharm. Teach. Learn..

[8] Mubarak N., Khan A.S., Zahid T., Aziz M.M., Khan R., Mahmood K., Saif-ur-Rehman N., Zin C.S. (2021). Assessment of Adherence to the Core Elements of Hospital Antibiotic Stewardship Programs: A Survey of the Tertiary Care Hospitals in Punjab, Pakistan. Antibiotics.

[9] PMC (2021). Pakistan Medical Commission. https://www.pmc.gov.pk/Colleges/PublicMedicalColleges.

[10] PCP (2021). Pharmacy Council Pakistan, Ministry of National Health Services Regulations & Coordination, Islaamabad. https://www.pharmacycouncil.org.pk/PI.php.

[11] Aziz M.M., Haider F., Rasool M., Hashmi F., Bahsir S., Li P., Zhao M., Alshammary T., Fang Y. (2021). Dispensing of Non-Prescribed Antibiotics from Community Pharmacies of Pakistan: A Cross-Sectional Survey of Pharmacy Staff’s Opinion. Antibiotics.

[12] Raheem M., Anwaar S., Aziz Z., Ali Raja S., Saif-Ur-Rehman N., Mubarak N. (2020). Adherence to the core elements of outpatient antibiotic stewardship: A cross-sectional survey in the tertiary care hospitals of Punjab, Pakistan. Infect. Drug Resist..

[13] Hayat K., Jamshed S., Rosenthal M., Haq N.U., Chang J., Rasool M.F., Malik U.R., Rehman A.U., Khan K.M., Fang Y. (2021). Understanding of Pharmacy Students towards Antibiotic Use, Antibiotic Resistance and Antibiotic Stewardship Programs: A Cross-Sectional Study from Punjab, Pakistan. Antibiotics.

[14] Abbo L.M., Cosgrove S.E., Pottinger P.S., Pereyra M., Sinkowitz-Cochran R., Srinivasan A., Webb D.J., Hooton T.M. (2013). Medical students’ perceptions and knowledge about antimicrobial stewardship: How are we educating our future prescribers?. Clin. Infect Dis..

[15] Saleem Z., Hassali M.A., Versporten A., Godman B., Hashmi F.K., Goossens H., Saleem F. (2019). A multicenter point prevalence survey of antibiotic use in Punjab, Pakistan: Findings and implications. Exp. Rev. Anti-Infect. Ther..

[16] Mubarak N. (2021). A multicentre point prevalence survey of the antibiotic use in tertiary care hospitals in Punjab. Antibiotics.

[17] Justo J.A., Gauthier T.P., Scheetz M., Chahine E.B., Bookstaver P.B., Gallagher J.C., Hermsen E.D., DePestel D.D., Ernst E., Jacobs D.M. (2014). Knowledge and Attitudes of Doctor of Pharmacy Students Regarding the Appropriate Use of Antimicrobials. Clin. Infect. Dis..

[18] Gyssens I.C. (2018). Role of Education in Antimicrobial Stewardship. Med. Clin. N. Am..

[19] HHsu J.L. (2018). Building an Antibiotic Stewardship Program: An Interactive Teaching Module for Medical Students. Mededportal.

[20] Shankar R., Bajracharya O., Jha N., Gurung S.B., Singh K.K. (2011). Teaching Medical Students to use Antibiotics Rationally in a Medical School in Nepal. Educ. Med. J..

[21] Antimicrobial Prescribing and Stewardship Competencies 2013. Department of Health, Expert Committee on Antibiotic Resistance and, Healthcare Associated Infections. https://assets.publishing.service.gov.uk/government/uploads/system/uploads/attachment_data/file/253094/ARHAIprescrcompetencies__2_.pdf.

[22] Efthymiou P., Gkentzi D., Dimitriou G. (2020). Knowledge, attitudes and perceptions of medical students on antimicrobial stewardship. Antibiotics.

[23] Saleem Z., Azmi Hassali M., Hashmi F., Azhar F., Mubarak R., Afzaal A., Munawar U. (2019). Medical and pharmacy students’ knowledge, attitude and perception concerning antimicrobial use and resistance in Pakistan. Pharm Educ..

[24] Hoque R., Mostafa A., Haque M. (2016). Insight of Medical Students of Clinical Years to Antimicrobials Prescribing and Resistance in Private Medical School, Chittagong, Bangladesh. J. Young-Pharm..

[25] Hassali M.A.A., Shafie A.A., Sulaiman S.A.S., Al-Haddad M.S.M., Tambyappa J., Masood I., Saleem F., Hari R., Nie C.G., Al-Qazaz H.K. (2011). Education on antibiotic resistance in medical and pharmacy schools: Findings from curriculum survey in selected southeast asian universities. https://www.reactgroup.org/uploads/react/resources/213/Education%20on%20antibiotic%20resistance%20in%20medical%20and%20pharmacy%20schools.en.266.pdf.

[26] Yang K., Wu D., Tan F., Shi S., Guo X., Min Q., Zhang X., Cheng H. (2016). Attitudes and perceptions regarding antimicrobial use and resistance among medical students in Central China. SpringerPlus.

