# Supplementary material for: How Are We Educating Future Physicians and Pharmacists in Pakistan? A Survey of the Medical and Pharmacy Student’s Perception on Learning and Preparedness to Assume Future Roles in Antibiotic Use and Resistance
Source: Antibiotics (Basel). 2021 Oct 3;10(10):1204. doi: 10.3390/antibiotics10101204 (PMC8532898; doi:10.3390/antibiotics10101204)
Supplement: Supplementary file 1 [file antibiotics-10-01204-s001.zip › antibiotics-1379536-supplementary.pdf]

Supplementary S1

a) **Demographics of respondents. (Table S1)**

b) **How well do you feel that your medical/ pharmacy education has prepared you to do the following upon graduation? (Table S2)**

a) (Table S1)

Table S1. Demographics of respondents.

	Medical Colleges n (%)			Pharmacy Colleges n (%)		
	**Public**	**Private**	**Total**	**Public**	**Private**	**Total**
**Institutes participated**	10 (38.5)	16 (61.5)	26 (57.8)	8 (42.1)	11 (57.9)	19 (42.2)
**Institutes Excluded**	-	-	4	-	-	20
**Response rate**	-	-	76.5%	-	-	70.4%
**Respondents**	175 (33.3)	351 (66.7)	526 (55.5)	193 (45.7)	229 (54.3)	422 (44.5)
**Male**	94 (53.7)	152 (43.3)	246 (46.8)	70 (36.3)	86 (37.6)	156 (37.0)
**Female**	81 (46.3)	199 (56.7)	280 (53.2)	123 (63.7)	143 (62.4)	266 (63.0)
**Complete rotation**	88 (50.3)	212 (60.4)	300 (57.0)	53 (27.5)	48 (21.0)	101 (23.9)

n= number, %= percentage.

b) (Table S2)

Table S2. How well do you feel that your medical/ pharmacy education has prepared you to do the following upon graduation?

Institute	Compared with	Institutes	Mean Difference	p-value
Medical Colleges Public		Medical Colleges Private	0.148	0.020*
		Pharmacy Colleges Public	0.020	0.783
		Pharmacy Colleges Private	0.047	0.496
Medical Colleges Private		Medical Colleges Public	-0.148	0.020*
		Pharmacy Colleges Public	-0.128	0.037*
		Pharmacy Colleges Private	-0.101	0.082
Pharmacy Colleges Public		Medical Colleges Public	-0.020	0.783
		Medical Colleges Private	0.128	0.037*
		Pharmacy Colleges Private	0.027	0.685
Pharmacy Colleges Private		Medical Colleges Public	-0.047	0.496
		Medical Colleges Private	0.101	0.082
		Pharmacy Colleges Public	-0.027	0.685

*p≤ 0.05 (significant difference between groups using One-way ANOVA.
